# Alginate's ability to prevent metabolic illnesses, the degradation of the gut's protective layer, and alginate‐based encapsulation methods

**DOI:** 10.1002/fsn3.4455

**Published:** 2024-10-02

**Authors:** Arslan Ahmad, Sakhawat Riaz, Derese Tamiru Desta

**Affiliations:** ^1^ Graduate School of Integrated Sciences for Life Hiroshima University Higashi‐Hiroshima Japan; ^2^ The State Key Laboratory of Tea Plant Biology and Utilization, School of Tea & Food Science Anhui Agricultural University Hefei China; ^3^ School of Nutrition, Food Science and Technology Hawassa University Hawassa Ethiopia

**Keywords:** alginate, Brown algae, gut health, intestinal barrier

## Abstract

The gut serves as the body's main immunological and digestive system. Furthermore, host immunity is mostly managed there. Nutrients are further broken down and absorbed here. Numerous research investigations have shown evidence that inflammation, oxidative stress, impairment of the intestinal barrier, and imbalance in the gut microbiota can all contribute to a variety of intestinal illnesses and other issues, underscoring the growing significance of intestinal health concerns. Because of their high biological activity and lack of negative side effects, the organic food‐derived chemicals known as brown algal polysaccharides—mainly *fucoidan, laminaran, and alginate*—have attracted a lot of attention from academics. Because of its many benefits, sodium alginate is one of the biopolymers that has garnered the greatest attention, research, and application. Carotenoids, which are found in our food, have several positive health effects. Their low solubility in water, oxidation susceptibility, chemical instability, accessibility, and restricted solubility in water, however, restrict their use in food and medicine. In this review and in overcoming these constraints, encapsulation might be helpful. Furthermore, the primary goal of our study is to provide a fresh perspective into the control and avoidance of intestinal diseases. It provides more potent alternatives to this system, clarifies the function of alginates in enhancing the encapsulation of carotenoids, and functions as a model for the synthesis of the intestinal protective agent fucoidan.

## Graphical abstract

The gut is crucial for digestion and immunity, with issues like inflammation and gut microbiota imbalance linked to intestinal diseases. Brown algal polysaccharides, particularly sodium alginate, offer promising benefits due to their high biological activity and minimal side effects. This review explores how encapsulation, particularly with alginates, can address challenges in using carotenoids and provides insights into fucoidan's role in intestinal protection.

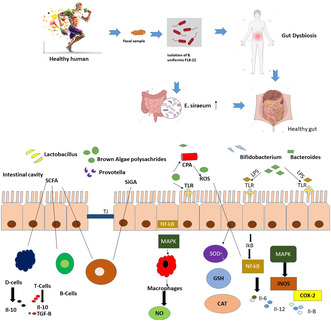

## 1 INTRODUCTION

Nutrients are processed down and digested in the gut, which is the body's largest immunological and gastrointestinal system. Apart from being able to identify, categorize, and distinguish dangerous illnesses from food items, immune cells in the stomach can work in tandem with bacteria to preserve the equilibrium of the gut ecosystem (Bain & Schridde, 2018; Yang & Yu, 2021). The digestive tract's chemical, physical, immunological, and microbial barriers keep endotoxins and infections from getting into the circulation and other tissues. On the other hand, intestinal barrier impairment can lead to entheogenic ailments, sepsis, inflammatory bowel disease, multiple organ dysfunction, and other diseases (Huo et al., 2022; Sauruk da Silva et al., 2021). A wide range of immunological, metabolism, or digestive tract issues have been linked to intestinal dysbiosis, according to numerous research studies (Bao et al., 2020; Hills et al., 2019; Liu et al., 2021; Ma et al., 2019; Tang & Cao, 2021).

The most prevalent substance discovered in the water was seaweed. According to their chemistry and color, they can be divided into three primary groups: brown, red, and green algae (Xu et al., 2017). According to Li et al. (2021), aerobic multicellular creatures and brown algae belong to the genera Macrocystis, Kelp, and Laminaria. Fucoidan, laminaran, and alginate are the principal polymers that brown algae make; these are also some of the principal active ingredients in brown algae (Chi et al., 2018; Dobrincic et al., 2020). Polysaccharides have been shown to have positive effects on the gut through the regulation of the microbiota in the gut, lessening of oxidative stress, stimulation of TJ protein expression, and suppression of the production of inflammatory molecules (Hwang et al., 2016; Iraha et al., 2013; Liu, Luthuli, et al., 2020; Xue et al., 2018). Alginates along with alginate hydrogels have garnered significant attention in recent years from a wide range of industries, including pharmaceuticals, food technology, biomedical engineering, and nanotechnology, among many others (Ching et al., 2017; Girón‐Hernández et al., 2021; Milivojevic et al., 2019, 2022; Sampaio et al., 2019). Alginates are great at retaining water and gelling, improving, and exhibiting rheological behavior, It enables them to be applied in a variety of contexts. They are often employed in the manufacturing of movies, pharmaceutical fibers, beads, and blends that include both organic and chemical polymers, as stated by Milivojevic et al. (2015).

Alginate is most commonly used in the food processing and medicinal industries. It is well recognized that chilling and dryness are the two primary unit processes utilized in this business for preservation. Nevertheless, when those techniques are applied to raw materials that contain certain chemical compounds, like carotenoids in addition, considerable losses in functionality and/or chemistry are usually observed (Aguirre Calvo & Santagapita, 2019). Carotenoids in fruits and vegetables, on the other hand, degrade easily (Souza et al., 2018); carotenoids within alginate hydrogel matrix, on the other hand, have improved biological stability and enhanced bioavailability (Soukoulis et al., 2016). Efficient encapsulation of active compounds, like carotenoids, requires careful selection of an appropriate encapsulating substrate and encapsulation process. These are frequently biopolymers that are suitable for food, including fats, proteins, carbs, and additional breakdown products that might support human physiology or structure. Polymers are the most widely used of them because of their excellent biodegradability, biocompatibility, affordability, and abundance (Debele et al., 2016). When encapsulating carotenoids, choosing an appropriate encapsulating agent is essential (Rostami et al., 2019). Several essential generic attributes that the encasing matrix must possess are the capacity to preserve expressed substances, the capacity to target and maintain release, the simplicity and strength of network development, the low cost and abundance, the proper dissolution, recyclable physical and chemical durability underperforming and conditions of preservation, and so on (Rehman et al., 2020).

This study investigates the mechanisms and health advantages of polysaccharides produced in the colon from brown algae. The key characteristics of carotenoids are outlined in this overview, along with the primary issues surrounding using them in a range of culinary and medicinal contexts. This paper examines the significance of carotenoid encapsulation and presents an extensive analysis of the many alginate‐based methods that are employed in this process, along with their respective advantages and disadvantages.

## 2 BROWN ALGAE POLYSACCHARIDES

Brown algae are rich in phenolic substances, sulfated polymers, quinones, and other additional metabolites, all of which have been investigated for potential medical use and nutritional advantages (Holdt & Kraan, 2011). Generally speaking, brown algal polysaccharides are defined as alginate, fucoidan, and laminaran, which are the three primary forms of polysaccharides that are separated and extracted from brown algae. The main species and geographic distribution of gut‐protective brown algae are shown in Table 1.

**TABLE 1:** The key kinds of brown algae with gut‐protective qualities and their geographical distribution.

Genus	Species	Distribution	References
*Macrocystis*	*Macrocystis pyrifera*	Primarily found along the ocean's beach in the northeast	Jia et al. (2022), Purcell‐Meyerink et al. (2021)
*Undaria*	*Undaria pinnatifida*	Endemic to China, Japan, and the Korean Peninsula; mostly from the northwest coast of the northern Pacific Sea	Jiang, Zheng, et al. (2021), Zhang et al. (2022)
*Laminaria*	*Laminaria japonica*	This species is primarily found in the northwest of the Pacific Coast. Extensively dispersed across China, Russia, Japan, and other countries	Fu, Zhan, et al. (2021)
*Fucus*	*Fucus vesiculosus*	Most often seen in warm or subtropical waters	Vodouhè et al. (2022), Wang et al. (2019)
*Ecklonia*	*Ecklonia cava*, *Ecklonia radiata*	Widely dispersed over China, Australia, Korea, and other nations; primarily found in the Chinese provinces of Shandong, Liaodong, Fujian, and Zhejiang	Lee et al. (2013), Charoensiddhi et al. (2016), Oh et al. (2021)

## 3 CLASSIFICATION OF BROWN ALGAE POLYSACCHARIDES

### 3.1 Alginate

**FIGURE 1:**
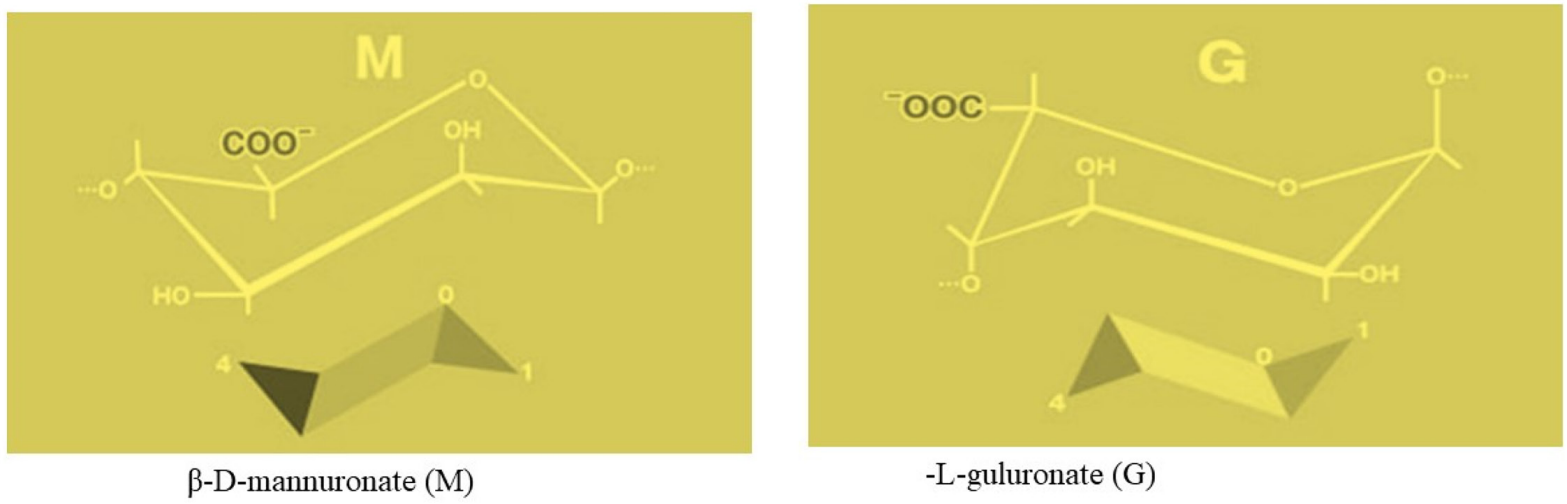
Structure of alginate.

The primary polymer found in brown algae's cell walls and intercellular matrix is alginate. The chemical composition of it is C6H7NaO6. Combining α‐L‐guluronate (G) and β‐D‐mannuronate (M), two conformationally isomer residues, results in a linear polysaccharide with 1,4‐glycosidic connections. Three distinct forms of the molecular structure are possible: MM, GG, and MG. Figure 1 indicates the alginate's molecular structure (Augst et al., 2006; Cong et al., 2014). The M/G ratio has a major impact on the chemical composition, applications, and properties of alginate (Sen, 2011). High G‐content chemicals produce hard gel for the pharmaceutical, foods, and cosmetics sectors, whereas high M‐content molecules provide a thin gel suitable for the production of paper, dyeing, nanoparticles, or the textile industry (Fawzy et al., 2017). Intestinal bacteria create short‐chain fatty acids fermenting alginate (SCFAs). These SCFAs are utilized by intestinal or immune epithelial cells as an energy source. Moreover, SCFAs affect how fats and carbohydrates are metabolized, which helps treat digestive disorders like diabetes, obesity, insulinemia, and other ailments (Gupta et al., 2019). Alginate therefore becomes crucial for preserving intestinal health and delaying the onset of metabolic disorders. Apart from its efficacious prevention of viral particle internalization by disruption of the connection among viral and host cell receptors (Salih et al., 2021; Serrano‐Aroca et al., 2021), SPMG has also been demonstrated to augment leukopenia generated by chemotherapy (Shi et al., 2020). Additionally, changing the composition of the intestinal microbiota, avoiding diet‐induced obesity, enhancing insulin sensitivity, and lowering inflammation raise the quantity of Lactobacillus and Bifidobacterium in the gut considerably (Liu et al., 2017).

One kind of polymer material that has thickening, gel‐forming, and stabilizing properties is alginate. Moreover, it is harmless, biodegradable, and biocompatible. High molecular weight and viscosity polymers make up alginate (Piras & Smith, 2020). It can improve glycemic control and encourage fullness because of its capacity to gel in the stomach and delay gastric emptying (Guo et al., 2020). Moreover, nutrients are better absorbed in the intestine when they remain in the stomach for a longer period of time as a result of delayed gastric emptying. Additionally, the intestinal mucosa may be shielded by a barrier formed by alginate's gelling property, which lowers inflammation and strengthens the intestinal barrier (Agüero et al., 2017). This allows the body to absorb and use healthy nutrients while preventing dangerous substances from passing through the stomach lining and into the circulation.

The relationships between intestinal cells and mucins as well as other nutrients and chemicals are impacted by alginate gelation, and this could have an effect on absorption and bioavailability (Brownlee et al., 2005). For example, the gelation of alginate may reduce the amount of dietary fat absorbed in the colon by binding to lipids and creating a complex that is ejected in the stool (Paxman et al., 2008). Those who have high blood cholesterol levels could benefit from this.

### 3.2 Extraction of brown algae polysaccharides

**FIGURE 2:**
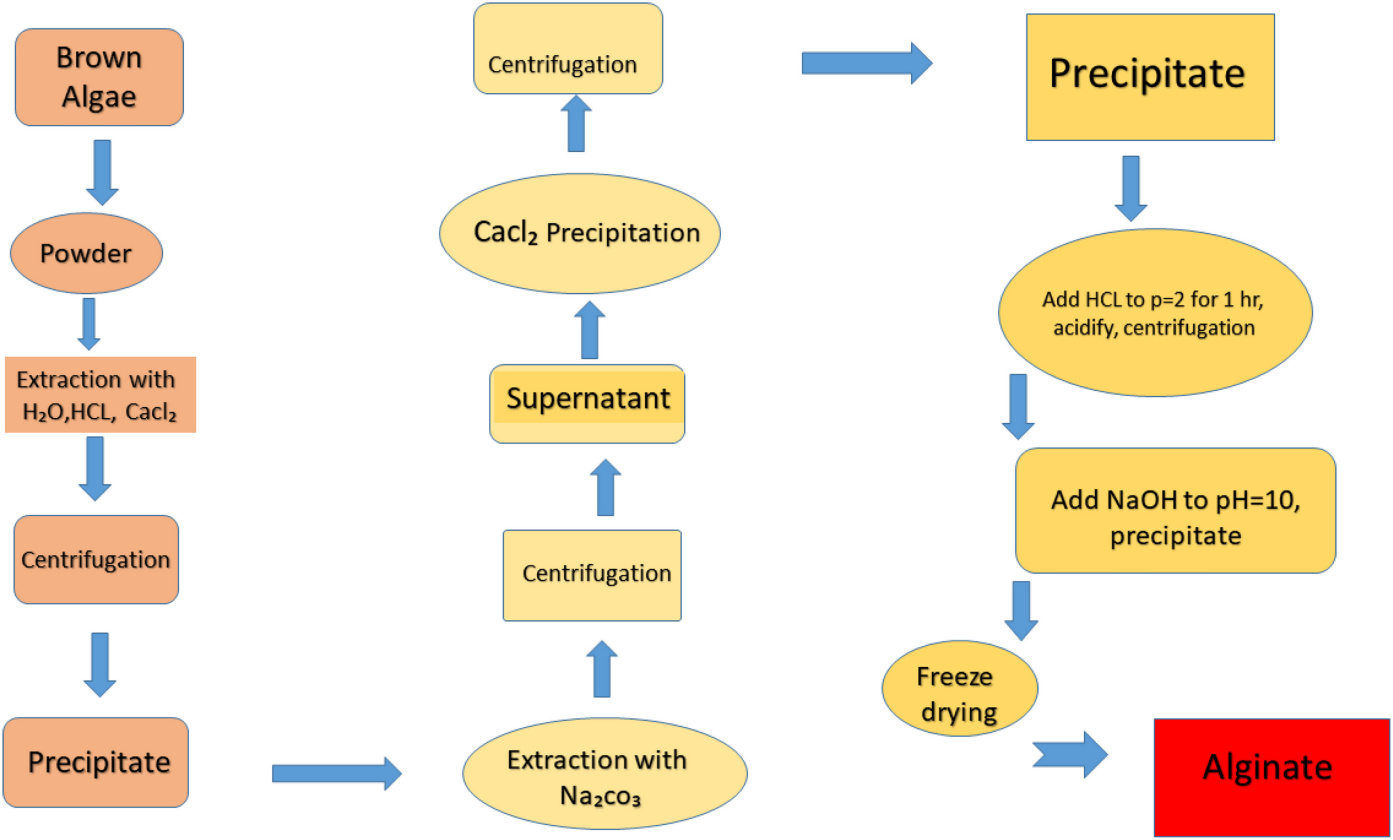
Flow chart of alginate extraction.

The conditions and methods of extraction can affect the molecular weight, yield, chemical composition, and biological activity of brown algal polysaccharide fractions (Zhao et al., 2020). Therefore, we need to choose the appropriate extraction procedures if we want to properly collect the necessary components. The stages associated with processing polysaccharides are disposal, extraction, and purifying. The chemical properties of polysaccharides can be used to select various extraction techniques, such as acid extraction, water extraction, auxiliary extraction, alkali removal, etc.; the molecular weight size of the polysaccharides can be used to select specific purification techniques, such as precipitation, column chromatography, etc. (Jia et al., 2020; Jiang, Yang, et al., 2021). Figure 2 illustrates the polysaccharide extraction method from brown algae (Abraham et al., 2019; January et al., 2019; Zhang et al., 2019).

### 3.3 Brown algae‐derived polysaccharides' protective effect on metabolic diseases and intestinal barrier damage

**FIGURE 3:**
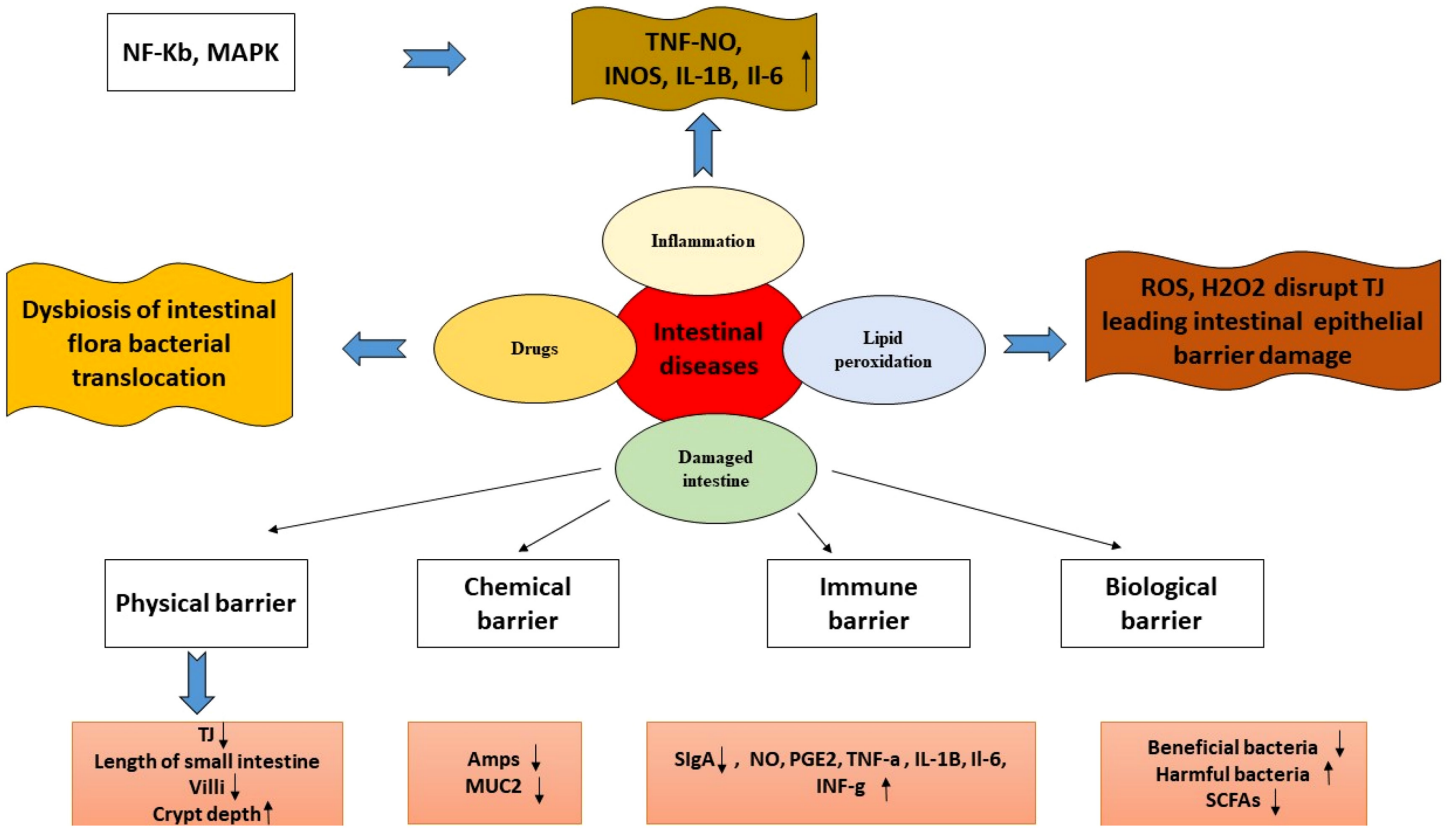
Pathogens of intestinal disease.

Intestinal health can be impacted by a multitude of factors, as illustrated in Figure 3. Lipid peroxidation, inflammatory processes, drug side effects, and intestinal barrier degradation are all potential causes of diseases linked to gut inflammation. T cells, B cells, and macrophages may release pro‐inflammatory cytokines such as TNF‐α, iNOS, NO, IL‐1β, COX‐2, and IL‐6. This secretion is triggered by the activation of nuclear factor kappa‐B (NF‐κB) and mitogen‐activated protein kinase (MAPK) pathways.

Reactive oxygen species (ROS) surplus production can cause immune cells to release pro‐inflammatory cytokines, leading to oxidative stress and inflammation. This further triggers signaling pathways such as PKC, MAPK, JNK, and ERK, which in turn impacts the functioning of the intestinal epithelial barrier by breaking down the TJ complex between epithelial cells (Bhattacharyya et al., 2014). Additionally, pharmaceutical treatment may impair the function of the gastrointestinal barrier (Mall et al., 2020).

The intestine protective characteristics of polysaccharides derived from brown algae are listed in Table 2. These polysaccharides regulate the microbiota of the gut, prevent inflammatory responses, and improve the production of transmembrane proteins by intestinal epithelial cells.

**TABLE 2:** The mechanism and preventive effect of polysaccharides generated from brown algae on the intestinal tract.

Source	Type	Inducer	Models	Function	Reference
*Laminaria*	Alginate	Cyclophosphamide	Mice	The synthesis of tight junction protein is increased Less harm to the gut mucosa Reduced expression of the mitogen‐activated protein kinase pathway, serum D‐lactate and lipopolysaccharide concentrations, and toll‐like receptor 4 to mitigate intestinal inflammation	Huang et al. (2021)
*Ascophyllum nodosum*	Alginate			Boosted the levels of acetate and propionate and encouraged the growth of Lactobacilli and Bifidobacteria	Jeong et al. (2017)
*Laminaria japonica*	Alginate			Bacteroides abundance increased	Ramnani et al. (2012)

### 3.4 Brown algae‐derived polysaccharides maintain intestinal barrier integrity

The components of the gastrointestinal barriers are chemical, physical, and immunological. The immune system activation brought on by deficiencies in barrier function over time may be harmful to several conditions, including obesity, diabetes, IBD, celiac disease, and colorectal cancer (Vancamelbeke & Vermeire, 2017). Intestinal wellness and disease prevention depend on maintaining the integrity of the intestinal barrier.

### 3.5 Preserving the structural integrity of the physical barrier

The gut's physical barrier consists of intercellular junctions, including adherens junctions (AJs), tight junctions (TJs), and bridging granules, as well as intestinal epithelial cells (IECs) (Branca et al., 2019). As the most intricate apical linker, the TJ is made up of internal plaque proteins like zonula occludens (ZO) and transmembrane proteins, including occludin, claudins, and junctional adhesion molecule (JAM) (Suzuki, 2020). The deterioration of the TJ, with its most significant intercellular structure, and increasing paracellular leakage are key players in the onset of IBD. Moreover, the measurement of the crypt's depth and the villi's length in the small intestine serve as significant markers for assessing small intestinal digestion and absorption. Enhanced absorption and digestion result in elongated villi and reduced crypt depth. Alginate oligosaccharides reduced inflammation‐induced damage in intestinal epithelial cells by upregulating occludin expression in TNF‐α‐treated IPEC‐J2 cells (Wan et al., 2020). As a result, by increasing the production of transmembrane proteins, triggering signaling pathways linked to the control of TJs, and reducing cellular permeability, polysaccharides generated from brown algae can enhance the damage to the interior physical barrier.

### 3.6 Enhancement of intestinal chemical barrier function

Proliferating mucins, saliva, bile acids, and antimicrobial peptides (AMPs) into the intestinal lumen, intestinal epithelial cells (IECs) create a variety of chemicals to prevent bacteria from adhering (Ding et al., 2021). Goblet cells can secrete mucins that harden into a gel to prevent bacteria from penetrating the intestinal mucosa (Zhang & Wu, 2020). MUC2 is the most common mucin in the colon and small intestine. Its absence can cause illness by altering mucus layers, affecting AMPs, and raising intestinal permeability, all of which can be pathways for harmful bacteria to enter the body and spread (Paone & Cani, 2020; Sicard et al., 2017). A.G. Smith discovered that supplementing the food with laminarin had a significant effect on the pig colon's synthesis of secreted MUC2 and membrane‐bound mucin MUC4. Smith et al. (2010) reported that the addition of polysaccharides derived from brown algae increased intestinal mucin synthesis. According to related research, polysaccharides' capacity to bind bile acids may be impacted by the sulfate groups present in them (Gao et al., 2017).

### 3.7 Improving intestinal immune barrier protection

The main element of the intestinal immune system, the body's greatest defense mechanism, is the lymphoid tissue of the gut (GALT), which accounts for 70% of peripheral immune function. The intestinal immune system consists of many kinds of immune cells, including T cells, B cells, innate lymphocytes (ILC1, ILC2, and ILC3), and a network of mononuclear phagocytes (monocytes, dendritic cells, and macrophages) (Ruder & Becker, 2020; Zhou et al., 2021). Tang et al. (2019) claim that these immune cells can regulate immunological responses and inflammation in the feces by releasing relevant pro‐ and anti‐inflammatory cytokines and enzymes. The intestinal mucosa's surface is home to lymphocytes and plasma cells, which are the primary producers of specific secretory immunoglobulins (sIgA). They are the immunoglobulins that are most prevalent in intestinal secretions and play a crucial role in the intestinal immune system by acting as the primary barriers to pathogen invasion.

Even though cyclophosphamide (CPA) is a frequently prescribed cancer treatment, long‐term usage of the drug can impair the immune system. Cancer is one of the main diseases that threaten human health (Xu & Zhang, 2015). According to Li et al. (2017), CPA causes apoptosis and dramatically raises ROS levels. Furthermore, it causes the spleen and thymus to produce fewer antioxidant enzymes, such as catalase (CAT), glutathione peroxidase (GSH‐Px), and superoxide dismutase (SOD). Additionally, in immunized mice, Laminaria sodium alginate has been shown to dramatically raise serum immunoglobulin and cytokine secretion, reduce splenic T cells, and improve immunological organ indices (Huang et al., 2021).

In TNF‐treated IPEC‐J2 cells, alginate oligosaccharides reduced TNF‐α and IL‐6 levels and prevented apoptosis (Wan et al., 2020). Pro‐inflammatory compounds have the ability, at least in certain concentration ranges, to stimulate the release of cytokines by other immune cells and to involve them in the immunological response, thus augmenting their antitumor efficacy. On the other hand, bioactive substances like NO and TNF‐α can potentially cytotoxically affect cells indirectly. So more research is required to determine if the bidirectional immune‐regulating impact of polysaccharides derived from brown algae might coordinate innate immunity and inflammatory responses by stimulating or inhibiting the release of cytokines associated with inflammation.

### 3.8 Brown seaweed polysaccharides' role in intestine

#### 3.8.1 The BSP's digestive behavior

Meals can absorb nutrients in the digestive tract, especially in the big bowel. The creation of an in vitro digestion model has provided us with a more comprehensive knowledge of the behavior of polysaccharides in the digestive system (Capuano, 2017). The primary subjects of discussion in the in vitro digestive system simulator are modifications to the molecular weight and free monosaccharides of polysaccharides.

The digestive tract consists of the mouth, esophagus, large bowel, stomach, and intestine. Several studies have demonstrated that naturally occurring polysaccharides can pass through the human stomach, small intestine, and salivary glands without being metabolized by the body's enzymes before entering the large intestine without any problems (Liu, Ma, et al., 2018; Flint et al., 2012). When polysaccharides were broken down in vitro, there was no change in the molecular weight of BSP or the quantity of free monosaccharides in the saliva, stomach, or small intestine.

Yet, the molecular weight and sugar concentration are significantly altered by the fermentation of gut microorganisms. Furthermore, it was discovered that the fermentation of human fecal samples was significantly impacted by variations in pH and SCFA level (Chen et al., 2018; Kong et al., 2016). When different polysaccharides are simulated to be digested in vitro, the outcomes are comparable (Gao et al., 2018; Hu et al., 2018; Xu et al., 2019). The precise breakdown mechanism of seaweed polysaccharides is unknown (El et al., 2013). Further research on the digestive habits of BSP is still required.

### 3.9 Brown algae's use in food and medicine

Brown seaweeds have high levels of proteins, carbs, and polyunsaturated fatty acids, while having low levels of lipids (Ferdouse et al., 2018). These are plants that have enzymes, minerals (both macro‐ and micronutrients), bioactive chemicals, and iodine (Imchen, 2021).

**FIGURE 4:**
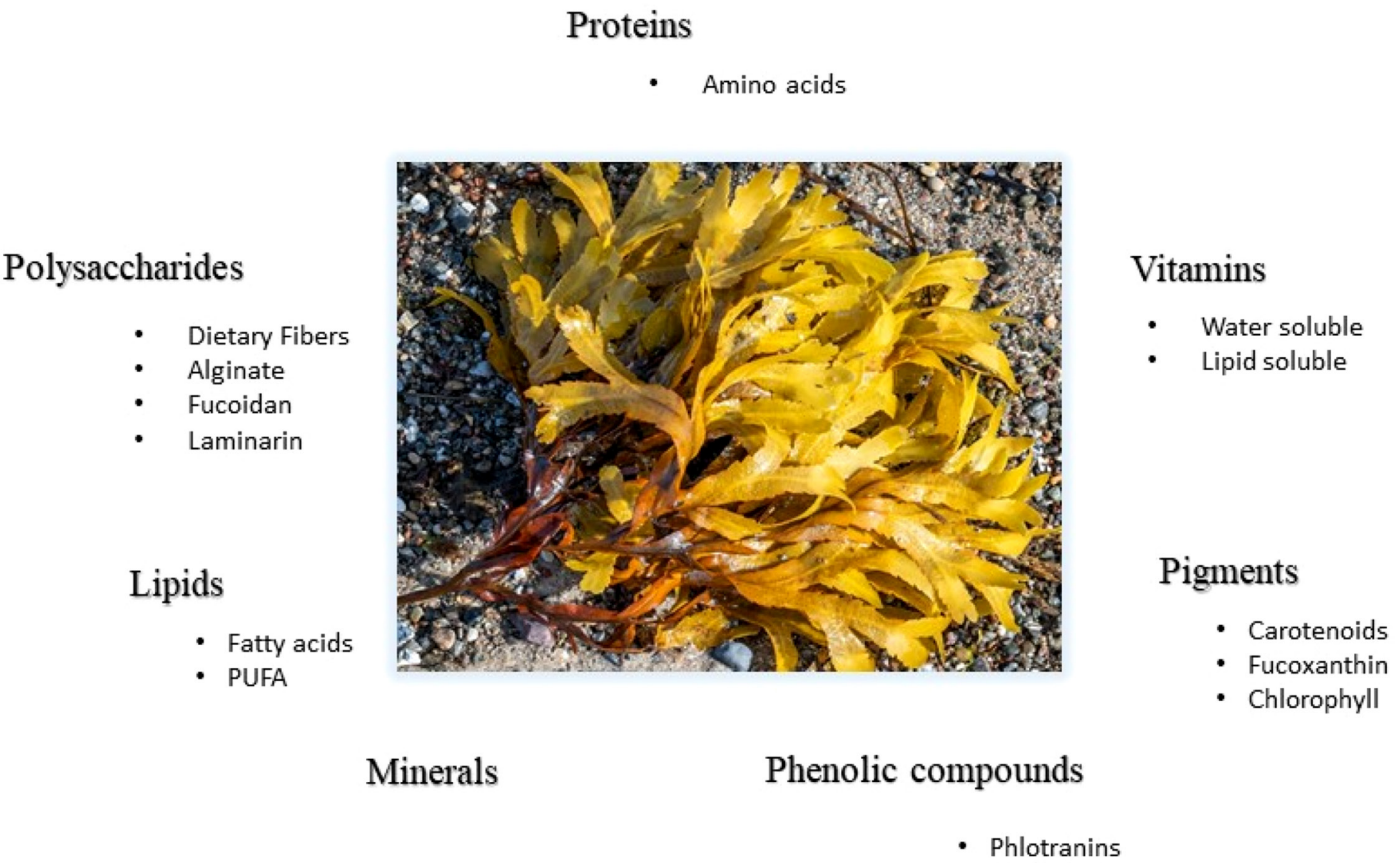
Bioactive components of brown seaweed.

Ghosh et al. (2022) and Jagtap et al. (2021) have found that brown seaweeds possess a unique chemical composition and contain a diverse range of bioactive chemicals (Figure 4), which have demonstrated physiological effects in both laboratory and living organisms. Minerals, including microelements, and organic materials, such as proteins, free amino acids, lipids (fatty acids), pigments, polyphenolic compounds, and spare carbohydrates like mannitol and laminarin, make up the majority of brown seaweeds.

**FIGURE 5:**
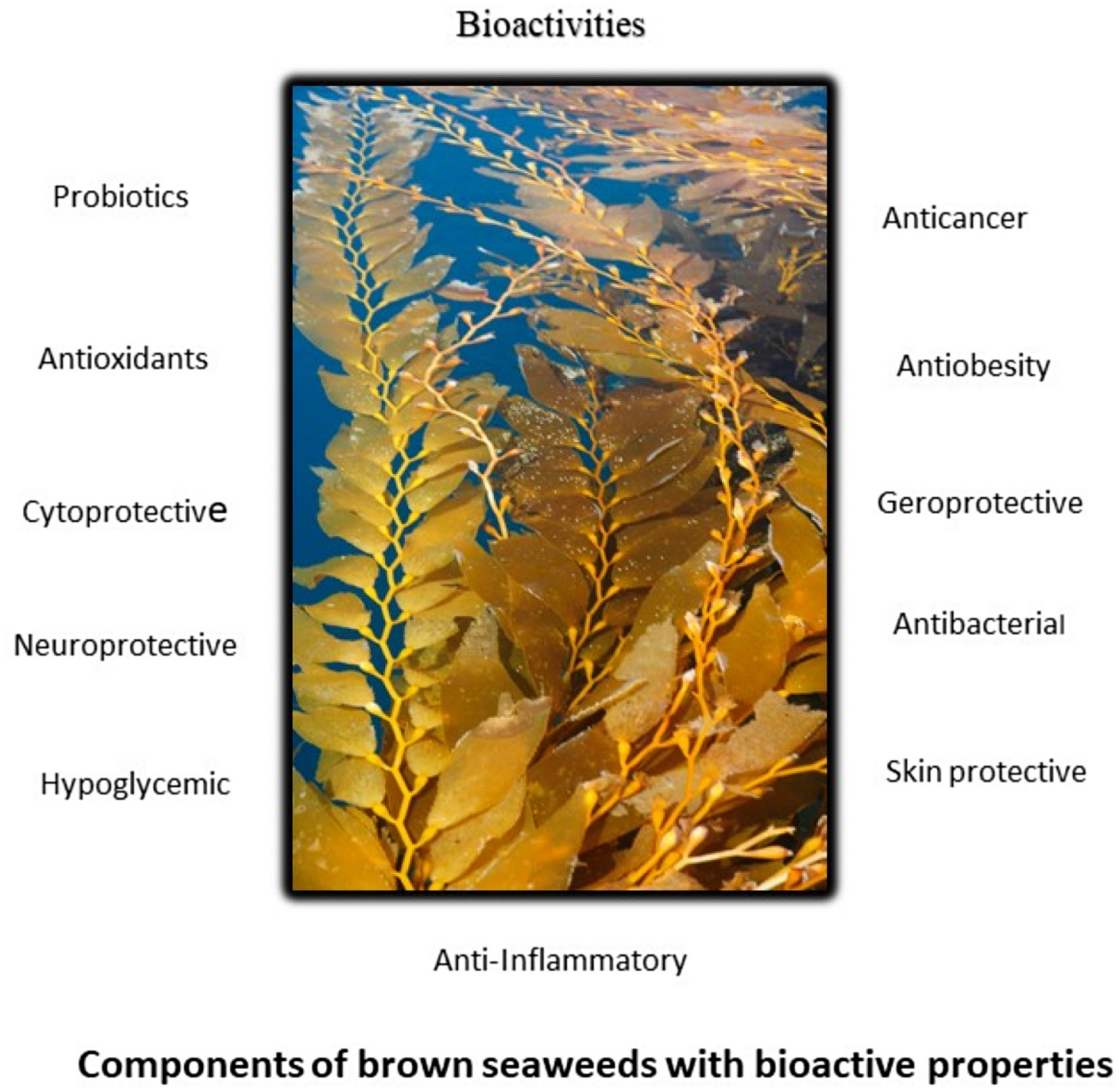
Bioactive properties of brown seaweed.

Due to their properties as antioxidants, anticoagulants, anti‐glycemic, antitumoral, and neuroprotectants, functional components extracted from brown seaweed have a great deal of potential in treating a variety of chronic diseases (Figure 5; Digala et al., 2022; Miyashita et al., 2020; Silva et al., 2019; Urrea‐Victoria et al., 2022). According to Ziyaei et al. (2022) and You et al. (2020), brown seaweeds must meet certain criteria before they can be utilized in nutraceuticals and medical applications.

### 3.10 Polysaccharides

Seaweed is a valuable source of nutrients because its polysaccharides are more diversified than those found in terrestrial plants (Bayu et al., 2021; Gullón et al., 2020). The majority of seaweed is made up of polymers, either sulfated or unsulfated. Brown seaweed has a high concentration of polysaccharides, including fucoidan, laminarin, and alginate, which are made up of monosaccharides, including mannose, xylose, fucose, rhamnose, glucose, and mannuronic and glucuronic acids (Reynolds et al., 2021). Clinical research suggests that bioactive substances obtained from seaweed may be used to treat and prevent COVID‐19. Moreover, seaweed‐derived sulfated polysaccharides and polyunsaturated fatty acids possess immunostimulatory and antitumorigenic properties (Ziyaei et al., 2022).

Alginates have extensive use in the food and pharmaceutical sectors for regulating blood glucose levels and mitigating gastrointestinal problems. Alginates lower energy expenditure and increase feelings of fullness without compromising normal function, according to human placebo‐controlled trials (Bermano et al., 2020; Wang et al., 2021). Due to its potent antitumor action against several cancer types, fucoidan, derived from brown seaweed, is the most well‐known anticancer medication (Sakthivel & Devi, 2019). Fucoidan is also used to treat and prevent the consequences of diabetes mellitus because of its hypoglycemic qualities (Wen et al., 2021). Alloyarova et al. (2024) state that fucoidan extracted from brown seaweed may be used in aquaculture as a valuable bioactive component in the diets of fish and shellfish.

### 3.11 Dietary fiber

The microbiota of the gut is crucial for maintaining overall health and preventing illness. Many ongoing studies aim to cure various diseases, including cancer, diabetes, and obesity, by using the gut microbiota to enhance the body's defenses (Clemente et al., 2012). It has been discovered that brown seaweed polymers are useful for inducing gut microbes. According to de Jesus Raposo et al. (2016), seaweeds make up 25–70% of all dietary fibers, of which 50–80% are soluble fibers.

Among other helpful bacteria, they are a nutritious substrate that promotes the growth of Bifidobacterium, Faecalibacterium, and Lactobacillus, as stated by Lopez‐Santamarina et al. (2020) and Deng et al. (2021). In vitro studies employing a human colon model have validated seaweed's prebiotic properties (Cronin et al., 2021). Dietary fibers function as prebiotics by boosting bacterial activity and triggering an immunological response in the gastrointestinal system.

This results in the synthesis of SCFAs, which have several beneficial physiological consequences. Seaweed has dietary fibers that have antiviral, antibacterial, coagulant, and antioxidant properties. Brown seaweed dietary fibers have extensive applications in food technology, mainly serving as thickeners, emulsifiers, gelling agents, and prebiotics (Pradhan et al., 2022).

### 3.12 Phenolic compounds

These are some of the seaweed's most significant bioactive constituents. Phlorotannins, tannins, flavonoids, phenolic acids, and catechins are a few of them. The kind of seaweed determines their composition. Phlorotannins, complex polymers consisting of phloroglucin linkages (1,3,5‐trihydroxybenzene), are the major component of brown seaweed. About 2 to 30% of the dry weight of seaweed is made up of polyphenols. It has been demonstrated that phenolic compounds in brown seaweed have antihyperglycemic and antihyperlipidemic properties (Mildenberger et al., 2022; Murray et al., 2018). Table 3 shows the pharmacological properties and potential uses of phenolic acids and polysaccharides via brown seaweeds.

**TABLE 3:** Therapeutic effects and applications of bioactive components of brown seaweed.

Seaweed genus	Active compound	Therapeutic impact	Applications	References
*Sargassum horneri*, *Sargassum hemiphyllum*, and *Sargassum vachellianum*	Polysaccharides	Antioxidant activity, tyrosinase, and elastase inhibition	Active ingredients that protect the skin	Jesumani et al. (2019)
*Eisenia bicyclis*	β‐Glucan (laminaran)	Stomach defense (in the event of gastric dysplasia)		Desamero et al. (2018)
*Fucus vesiculos*, *Ascophyllum nodosum*	Sodium alginate	Enhancement of the human intestinal microbiome	Prebiotics	Sokolan and Kuranova (2021)
*Padina boryana*, *Turbinaria ornate*, and *Sargassum polycystum*	Fucoidan, laminaran, and alginate	Activity of antioxidants	Useful components	Mohd Fauziee et al. (2021)
*Padina tetrastromatica*	Fucoxanthin, lipids	Possessing anti‐inflammatory properties	Beneficial elements	Sharma et al. (2023)
*Ascophyllum nodosum*	Phlorotannins	Reduced DNA damage in fat people	Additives with biological activity	Baldrick et al. (2018)
*Ascophyllum nodosum*, *Fucus vesiculosus*	Polyphenols	Preventing type 2 diabetes	Bioactive additives	Vodouhè et al. (2022)
*Laminaria japonica*	Fucoidan	Immunostimulating, antitumoral, and anti‐inflammatory properties	Medicines, adjuvants	An et al. (2022)
*Ecklonia kurrome*, *Hizikia fusiforme*, and *Undaria pinnatifida* Suringar	Phenolic acids, flavonoids	Treatment for diabetes: α‐glucosidase inhibitors	Medicines	Xie et al. (2021)
*Fucus evanescens*		Effects of antioxidants and hepatoprotection	Functional foods (bread)	Fedyanina et al. (2018)

Phloro tannins have antitumorous, cytotoxic, anti‐inflammatory, antimicrobial, and antioxidant properties (Kaushalya & Gunathilake, 2022). Additionally, they might be employed as anti‐aging substances (Obluchinskaya et al., 2019).

### 3.13 Brown seaweed applications in food technology

Brown seaweeds include S. japonica, F. evanescens, L. digitata, S. latissima, and U. pinnatifida. The food industry mostly uses these seaweeds (Milinovic et al., 2021). Seaweed may be harvested from its natural habitat or cultivated on dedicated aquaculture facilities. The global production of brown seaweed had a significant growth from 13,000 tons to 16.4 million tons between the years 1950 and 2019. From 1950 to 2019, its average yearly increase exceeded the worldwide expansion of aquaculture for all species combined. Cai et al. (2021) reported that *Laminaria saccharina* and *U. pinnatifida*, two types of brown seaweeds, constituted 47.3% of the total worldwide seaweed production in 2019.

In order to promote health and lower the incidence of dietary‐related illnesses, brown seaweeds are frequently included as functional ingredients in food manufacturing (Ghosh et al., 2022). Brown seaweeds have biological activity in addition to their physical and chemical characteristics, which makes them useful for nutraceuticals. Table 4 displays the functional roles and uses of the bioactive components of brown seaweeds in food items. There is a growing trend in food technology to employ brown seaweeds and their constituents, such as protein extracts and polysaccharides (Mouritsen et al., 2019). Functional foods, especially those derived from seaweed, are gaining popularity in several regions where the use of seaweed is prohibited due to several reasons (Blikra et al., 2021).

**TABLE 4:** Functional roles and applications of bioactive components of brown seaweeds in food product.

Component	Functional role	Food product	References
Sodium alginate	Structural modifier for 3D printing	Paste made of rice (3D printing)	Liu, Tang, et al. (2020)
Sodium alginate	Modulator for structure in 3D printing	3D‐printed artificial steak	Tsai and Lin (2022)
*Ascophyllum nodosum*, *Fucus vesiculosus*, and *Bifurcaria bifurcata* aqueous extracts	Dietary antioxidant	Pork liver pâté	Hoffmann et al. (2021)
*Himanthalia elongata*, dried and shred (phenolic chemicals)	Nutritional antioxidant	Cheese	Boutheina et al. (2023)
Fucoidan	A cell inhibitor for oral cancer	Shake	Kumar et al. (2019)
Fucoidan (*Fucus vesiculosus*)	Antimicrobial substance	A useful pasteurized apple drink	Kang et al. (2020)
*Alaria esculenta*	Enhancer of nutritional value and gelling agent	Fillings made with pastry	Krekhnova et al. (2019)

### 3.14 Preservation of the intestinal microbial barrier via the equilibrium of the intestinal microbiota

The gut microbiota plays a crucial role in protecting the host's health by controlling the growth and functioning of the immune system, regulating metabolism, preserving the integrity of the epithelium, and reducing the likelihood of weight gain and severe diabetes (Artis, 2008; Li et al., 2020; Luthuli et al., 2019; Vandana et al., 2020). In general, polymers increase the gut microbiota's beneficial effects and facilitate the passage of digestible polysaccharides into the colon for fermentation, which in turn promotes the development of microorganisms that create SCFAs.

The immune system and gut microbiota become dysregulated when this mutual equilibrium is disrupted, increasing the body's vulnerability to pathogenic infections and resulting in a range of disorders (Spiljar et al., 2017). In order to preserve the well‐being of the host, the immune system and gut microbiota often collaborate to regulate the body's immunological response and metabolic processes (Ahmad, Riaz, & Tanveer, 2022). Bacteroides are less abundant in the gut microbiome of IBD patients than Proteus, which includes bacteria like Clostridium and Escherichia coli. Thicker mucus layers result from decreased Paneth and Goblet cell counts and functions, which worsen mucosal integrity, encourage gastrointestinal dysbiosis, and eventually cause bacterial translocation and impaired intestinal physical barrier function (Sommer et al., 2017). To raise the intestinal crypt depth ratio, fucoidan and laminaran, both produced from brown algae, were fed to weaned pigs. According to Walsh et al. (2013), there was a significant reduction in the amount of Enterobacteriaceae in the colon, along with a drop in the activity of IL‐6, IL‐17A, and IL‐1β mRNA. Moreover, polysaccharides promote the proliferation of bacteria that synthesize SCFAs, thus promoting the well‐being of the gut microbiota. The gut undergoes anaerobic fermentation of polysaccharides, leading to the synthesis of SCFAs by bacteria. This process increases anaerobic metabolism and subsequently lowers the pH of the colon, therefore inhibiting the development of dangerous gram‐negative bacteria (Blaak et al., 2020).

Alginate oligosaccharides increased the presence of Firmicutes and Actinobacteria while reducing the proportion of Bacteroidetes bacteria in the colons of mice with ulcerative colitis. The findings indicate that alginate fatty acids effectively regulated the intestinal environment, hence preserving the integrity of the mucosal barrier (He et al., 2021). Similarly, the molecules produced by the microorganisms in the intestines, known as intestinal microbiota compounds, or SCFAs, have the ability to enhance blood lipid levels, reduce inflammation and insulin resistance, regulate digestion, and influence blood glucose levels by specifically affecting the pancreas, liver, intestines, and other organs (Schoeler & Caesar, 2019; Liu et al., 2022). SCFAs provide energy to intestinal cells and modify both adaptive and innate immune responses, in addition to reducing the risk of inflammatory diseases (Martin‐Gallausiaux et al., 2021; Rooks & Garrett, 2016). Research has shown that the process of fermenting sodium alginate from *Ascophyllum nodosum* in a laboratory setting may substantially increase the levels of acetate and propionate (Ramnani et al., 2012). Sargassum fusiforme alginate enhances the activity of antioxidant enzymes (CAT, SOD) in the bloodstream, hence mitigating oxidative stress in rats subjected to a high‐fat diet with the purpose of inducing diabetes. Therefore, polysaccharides obtained from brown algae may shield the gut from damage caused by oxidative stress by reducing the production of ROS or enhancing the activity of antioxidant enzymes.

## 4 THE EFFECTS ON THE GUT MICROBIOTA OF BSP

To evaluate the effect on the gut microbiota, measurements of the amount of SCFAs, the composition, and the quantity of beneficial intestinal bacteria are commonly employed (Flint et al., 2008).

### 4.1 SCFAs production

As stated by Flint et al. (2008), SCFAs, such as acetate, propionate, and butyrate acids, are the primary byproduct of gut bacteria's fermentation of polysaccharides. As the main energy source for host cells to produce glucose and lipids, they are quickly absorbed and metabolized by the host (Yang et al., 2013). Research suggests that SCFAs may be essential for improving gastrointestinal diseases, cancer, and metabolic disorders (Donohoe et al., 2011). When taken orally, acetate acid can improve glucose tolerance and prevent obesity. It is essential for the synthesis of cholesterol.

Bacteroides, Phascolarctobacterium, Dialister, and Veillonella's principal metabolite is propionic acid, which can control the levels of insulin and blood sugar to prevent diet‐induced obesity (Gao et al., 2009). The most popular route for synthesizing propionate is the succinate pathway, which begins with the production of oxaloacetate from pyruvate, then transforms to succinate, succinyl‐CoA, propionyl‐CoA, and finally propionate (Louis et al., 2014). Firmicutes' primary metabolite, butyrate acid, controls gut hormones and reduces swelling in the gut (Lin et al., 2012).

In accordance with Correa et al. (2016), SCFAs can activate intestinal epithelial cells, which are capable of synthesizing mucus and antibacterial substances. In order to produce metabolites (SCFAs) that activate relational gene expression, bacteria like Lactobacillus, Bifidobacterium, Roseburia, Faecalibacterium, Anaerostipes, Coprococcus, and others can digest brown seaweed polysaccharides (BSS) in the large intestine (Fernandez et al., 2016). Therapeutic strategies for type II obesity and diabetes may include SCFAs. As byproducts of the digestion of polysaccharides found in brown seaweed, they are important in preserving the wellness of the organism in question and preventing illness.

### 4.2 Optimizing the gut microbiota's development and activity

By using bacterial 16S ribosomal RNA (16S rRNA) sequencing by fermenting feces, brown seaweed polymers have the ability to alter the makeup of the gut microbiota (An et al., 2013). Most types of bacteria are typically assessed using feces via in vitro colon studies (Roberfroid, 2007). According to research, two phyla of bacteria, Firmicutes and Bacteroidetes, make up over 90% of the colonic microbiota. Shang et al. (2016) discovered that the gut microbiota could be modulated by Enteromorpha Clathrata polysaccharide, resulting in a significant rise in the development of friendly bacteria (*Bacteroides*, *B. thetaiotaomicron*, *B. distasonis*, and *B. fragilis*) and a substantial reduction in the development of harmful bacteria (*Firmicutes*, *Alloprevotella*, and *Blautia*). Additionally favorable to gut health are Firmicutes and Bacteroidetes (Mariat et al., 2009). Typical gut microbiota that promote health also includes Lactobacillus, Anaerostipes, and Bifidobacterium (Fernandez et al., 2016). Akkermansia muciniphila, a newly developed probiotic bacteria, has recently been associated with metabolic disease and obesity.

The microbial composition of mice given a high‐fat diet supplemented with fucoidan produced from *Ascophyllum nodosum* and *Laminaria japonica* showed an increase in the proportion of SCFA producers, including Akkermansia muciniphila, Alloprevotella, Blautia, and Bacteroides (Shang et al., 2017). By boosting the numbers of varieties of Bifidobacterium and Akkermansia muciniphila in female mice, enteromorpha clathrate polysaccharides may alter the gut flora. According to You et al. (2020), brown seaweed polymers may have prebiotic activity by altering the makeup and boosting the quantity of gut bacteria.

### 4.3 The intestinal epithelium and BSP

The fundamental building block for preserving an effective intestinal barrier is the intestinal epithelium that is adhered to the mucus layer. The equilibrium between the regrowth and mortality of intestinal epithelial cells is broken, and mucosal integrity is compromised when microorganisms, inflammation, toxic luminal substances, and oxidative stress disturb intestinal cellular homeostasis. This leads to a variety of intestinal diseases, including enteric infections and IBD (Wells et al., 2017).

For maintaining the antioxidant status of cells and encouraging the generation of cytokines, BSP affects intestinal epithelial cells. *Undaria pinnatifida* fucoidan protected swine intestinal epithelial cells IPEC‐1 from oxidative damage caused by hydrogen peroxide (H_2_O_2_). This could aid in the development of suitable protocols for preserving the gut health of young pigs (Li et al., 2019).

Reactive oxygen species, also known as ROS, were produced by H_2_O_2_, which quickly permeated the cell membrane and reacted with intracellular transition metals to produce extremely lethal hydroxyl radicals. The IPEC‐1 cells' antioxidant system was also harmed by the excessive generation of ROS. By boosting glutathione levels in cells and reducing malonaldehyde buildup, fucoidan significantly lowered ROS production while maintaining the antioxidant status of the cells. This advantageous effect was facilitated by the regulation of the nuclear factor erythroid 2‐related factor 2 (NRF2) signaling pathway. Nuclear transcription factor NRF2, which is widely expressed in various cell types, is responsible for regulating a group of defense genes that generate antioxidant proteins and detoxification enzymes. By promoting NRF2's translocation to the nucleus in cells, fucoidan administration dramatically raised the extent of nuclear fluorescence. Overall enhanced antioxidant status and decreased intracellular ROS generation of IPEC‐1 cells were in line with the rise in nuclear NRF2 activity.

Additionally, NRF2's downstream target genes, including NQO1, SOD1, and GPX1, had higher mRNA levels after fucoidan treatment. In order to lessen the oxidative stress that intestinal epithelial cells endure, Figure 2 shows how BSP controls the NRF2 signaling pathway (Sanjeewa et al., 2021). BSP has been shown in recent studies to reduce intestinal inflammation and stop the production of proinflammatory mediators. Sargassum hemiphyllum fucoidan significantly decreased gut inflammation in Caco‐2 cells by upregulating anti‐inflammatory cytokines like IL‐10 and IFN‐γ and downregulating proinflammatory mediators like TNF‐α and IL‐1β (Hwang et al., 2016).

Sargassum fusiforme polymers enhanced the integrity of the intestinal barrier and reduced LPS‐induced inflammation in IEC‐6 cells by suppressing the expression of TLR‐4, TNF‐α, IL‐6, IL‐1β, and inducible nitric oxide production, as well as inhibiting the synthesis of IL‐10 (Yao, Liu, et al., 2022). An animal study showed that consumption of fucoidan improved the function of the intestinal barrier by boosting the expression of TJ proteins and reducing plasma endotoxin levels via the activation of p38 MAPK and ERK1/2 phosphorylation (Xue et al., 2018). Supplementing with BSP resulted in an increase in the number of goblet cells that contain mucin in the middle and proximal intestine. Additionally, there was a substantial rise in the levels of mucins, namely the secreted mucin MUC2 and the membrane‐bound mucin MUC4 (Smith et al., 2010). According to Mirshafiey et al. (2005), alginate that has the right viscosity has also been used as a novel treatment alternative for treating experimental colitis. Despite not being able to be absorbed in the small intestine, the viscosity of sodium alginate allows it to stick to the mucosal surface. That adhesive reduces intestinal swelling and harm by preventing intestinal bacteria from penetrating the small intestine. This finding was in line with MUC1‐4's downregulated mRNA expression. The identical results were seen in the investigation using a mouse model of severe tissue damage (Horibe et al., 2016).

### 4.4 BSP and gut microbiota

In order for indigenous microorganisms to ferment and use BSP, it must first pass through the distal intestinal system before they can be effectively digested and absorbed via the abdominal wall. According to Nicholson et al. (2012), the gut microbiota produces interaction between host–microbiota metabolism, signals, and the immune‐inflammatory axis by regulating various host metabolic pathways. Recent research shows that BSP and its metabolites enhance the diversity and quantity of gut microbiota through fermentation, as shown in Table 1. The bacteria in the gut are regulated differently by laminarin, fucoidan, and alginate. It has been demonstrated that laminarin exerts prebiotic effects on gut microbiota, which means that it can favorably stimulate the development of good bacteria. Research has revealed that laminarin enhances the quantity of Bifidobacteria and Lactobacillus in the digestive tract; both of them are recognized to offer numerous health benefits (Vigors et al., 2020). The study by Ahmad et al. (2021) shows that fucoidan has anti‐inflammatory properties in the gastrointestinal tract, which might potentially reduce inflammation and improve gut health. Alginate is mostly known for its ability to create gels that adhere to various things in the gastrointestinal tract. However, it has also been shown to have some prebiotic effects on the gut bacteria (Ramdhan et al., 2020). It could limit certain nutrient absorption as well as lengthen the time food takes to pass through the stomach.

Prebiotic action can be proven, according to Yao, Gong, et al. (2022) & Ou et al. (2022), by creating beneficial chemicals such as SCFAs and by actively enhancing the growth of beneficial bacteria or increasing their relative abundance. One of the most often used techniques for studying prebiotic action is in vitro fecal fermentation. *Ascophyllum nodosum* polysaccharides were shown to enhance the levels of Bacteroidetes and Firmicutes, as well as boost the total production of SCFAs, via in vitro human fecal fermentation (Chen et al., 2018). The chemicals further augmented the levels of butyric acid, acetic acid, propionic acid, and overall SCFAs throughout the process of in vitro fermentation. Kong et al. (2021) found that fermenting Sargassum fusiforme polysaccharides led to a notable increase in the levels of Faecalibacterium, Phascolarctobacterium, Bifidobacterium, Ruminococcaceae_UCG‐014, and Lactobacillus. At the same time, it significantly reduced the levels of Prevotella_9, Blautia, and Prevotella_2. Additionally, it stimulated the synthesis of SCFAs such as acetic acid, propionic acid, and n‐butyric acid.

The quantity of SCFAs and the microbiological pathways related to the growth of bacteria that make SCFAs, including Lactobacillus, Bacteroides, Bifidobacterium, Streptococcus, and Bacillus, enhanced when BSx1P was supplemented. During fucoidan administration, rats with breast cancer displayed elevated gut microbiota diversity, a greater Bacteroidetes to Firmicutes phylum ratio, and elevation of the SCFA‐producing bacteria Prevotella (Xue et al., 2018). Alginate is a digestible food fiber that some bacteria in the human gut may be able to utilize (Fu et al., 2023; Fu, Pan, et al., 2021). Previous research indicated that the colon's health advantages might be attributed to the alginate‐fermenting bacterium Bacteroides xylanisolvens AY11‐1. In addition, when orally delivered, B. xylanisolvens AY11‐1 provides protection to mice against ulcerative colitis induced by dextran‐sulfate‐sodium (DSS). When given to mice via their food, B. uniformis F18‐22, a bacterium that ferments alginate and is found in a healthy human colon, has the ability to prevent DSS‐induced UC (Dai et al., 2023).

## 5 INHIBITION OF INFLAMMATORY CYTOKINES BY BROWN ALGAE‐DERIVED POLYSACCHARIDES

An atypical immune response provides protection against and repels infections, while an immunological reaction increases the vulnerability of the intestines to them. Tang et al. (2019) claim that discomfort is a typical immunologic reaction in living organisms. Evidence demonstrates that brown algae synthesize polysaccharides that inhibit the secretion of cytokines responsible for inducing inflammation. Sargassum horneri fucoidan demonstrated a dose‐dependent reduction in protein expression of iNOS and COX‐2, phosphorylation of ERK1/2 and p38, and production of TNF‐α and IL‐1β in LPS‐treated RAW 264.7 cells (Sanjeewa et al., 2017). Due to their ability to suppress the NF‐κB and MAPK pathways and the generation of related inflammatory proteins, brown algae‐derived polysaccharides may be used as effective calming meals.

**FIGURE 6:**
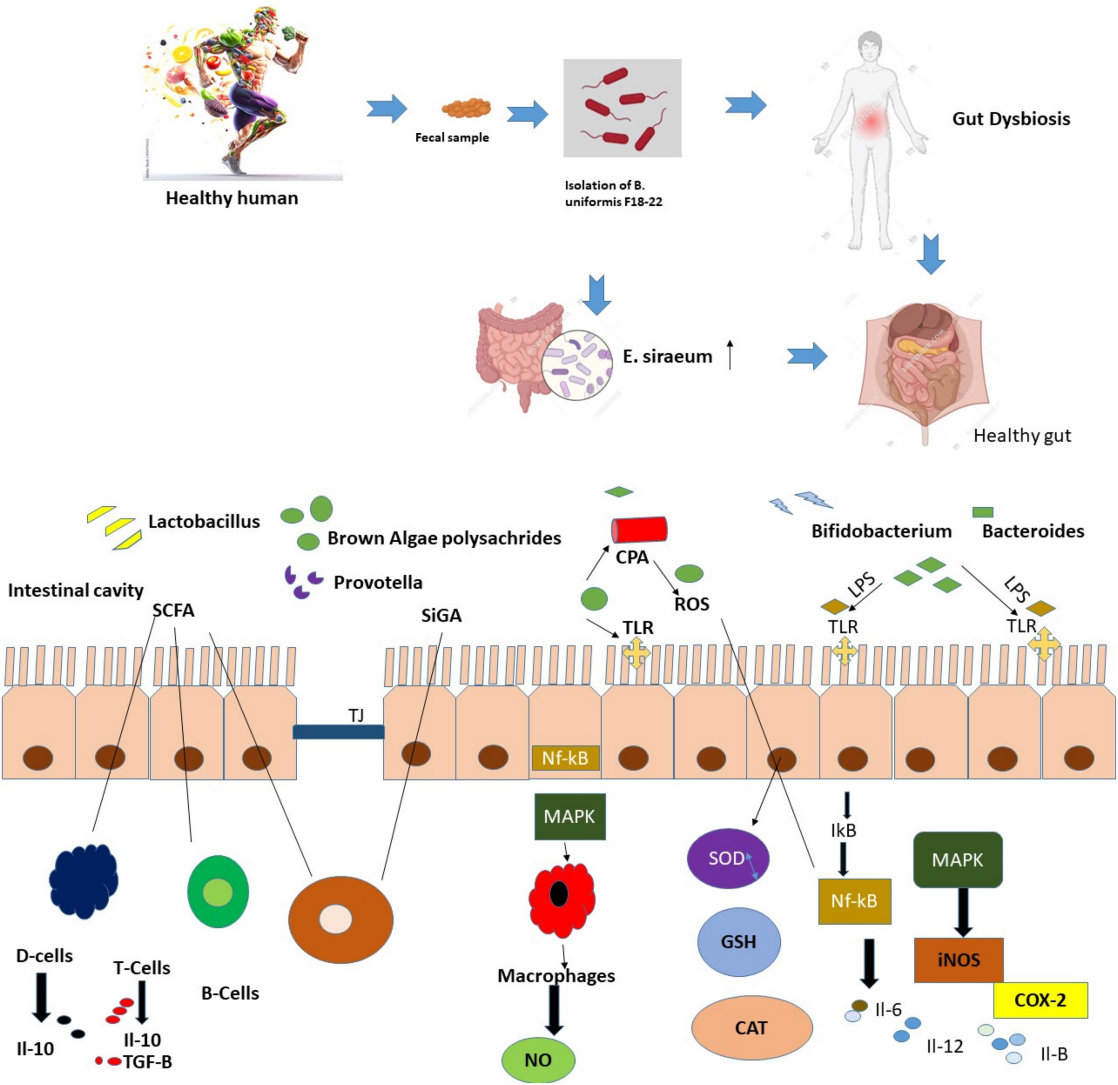
Defensive mechanisms of polysaccharides generated from brown algae against harm to the intestines.

Figure 6 illustrates the preventive effect of polysaccharides generated from brown algae against intestinal injury. Prebiotics generated from brown algae can enhance the production of the TJ protein to preserve the intestinal wall's integrity, hence decreasing pathogen invasion and bacterial translocation. Brown algae generated polysaccharides that boosted the number of helpful intestinal bacteria, controlled the microbiota's diversity, and encouraged goblet cells to create AMP and SCFAs in addition to MUC2. Additionally, they strengthened the intestinal microbiological barrier and improved intestinal resistance by improving the function of the intestinal chemicals barrier and inducing the release of anti‐inflammatory cytokines and antibodies by DC, T, and B cells. Brown algae secrete polysaccharides that have the ability to activate the NF‐κB and MAPK signaling pathways, hence enhancing the immune response. This may enhance the production of inflammatory factors and counteract the immunosuppressive effects of the chemotherapeutic medication CPA. The polysaccharides prevented the activation of the NF‐κB pathway, hence inhibiting the formation of reactive oxygen species. The following stimulated the production of enzymes that safeguard cells and reduce the inflammation caused by LPS. In summary, polysaccharides inhibited the activation of NF‐κB and MAPK signaling networks, resulting in the inhibition of inflammatory cytokines and the generation of beneficial characteristics.

### 5.1 BSP and their function in the intestinal barrier

The stomach and intestinal enzymes were unwilling to break down and digest BSP, but certain gut bacteria were able to do so. The composition of the microbiota was altered by the BSP intervention. Metabolite production from BSP fermentation included SCFAs. Because they are easily absorbed, SCFAs maintain the integrity of the intestinal barrier and the immune system. BSP preserved intestinal integrity by promoting the production of cytokines, preserving intestinal permeability, and upholding cellular antioxidant state (You et al., 2020). When administered dietaryly to mice, B. uniformis F18‐22, an alginate‐fermenting bacterium from a healthy human colon, can stop DSS‐induced UC (Dai et al., 2023).

### 5.2 Carotenoids

The red, orange, and yellow hues of vegetables and fruits are caused by terpenoid pigments called carotenoids, which are produced by a variety of plants, fungi, and bacteria. Lutein, zeaxanthin, and β‐carotene are among the most prevalent and significant of the over a thousand naturally occurring carotenoids used in food and medicine. Although humans can convert some carotenoids (β‐, β‐, γ‐carotene and α‐, β‐cryptoxanthin) into a material known as vitamin A, zeaxanthin, lutein, and lycopene are not known to have any vitamin A activity (Toragall et al., 2020). The physical features of carotenoids, such as color, light absorption intensity, molecular structure, chemical reactivity, and antioxidant activity, are determined by the length of the conjugated and delocalized electron system in the polyene chain (Dutta et al., 2011). Carotenoids are an essential component of pigment‐protein interactions found in the thylakoid membranes of chloroplasts and are involved in photosynthesis. In both photosystems I and II, such membranes are connected to the antenna complex and reaction centers. Carotenoids, as opposed to chlorophyll molecules, can absorb light with wavelengths between 460 and 550 nm, which is necessary to initiate the basic sunlight reaction paths of photosynthesis. Approximately half of the more than a thousand carotenoids found in nature are involved in the task of absorbing light during photosynthesis (Polívka & Frank, 2010). Carotenoids have a role in how plants respond to environmental stress because they may scavenge ROS in addition to their functions in light harvesting in chloroplasts and photoprotection in situations of abundant light (Green & Parson, 2009). Stressful environments release singlet oxygen and ROS, which break down carotenoids into a variety of aldehydes, endoperoxides, ketones, and lactones. These incredibly reactive electrophile materials can alter gene expression and trigger reactions to external stimuli (Havaux, 2014).

According to Debele et al. (2016), carotenoids are mostly utilized as food components as antioxidants, pigments, and flavor boosters. Because of their abundance of conjugated double bonds, carotenoids provide many other health benefits for humans, but their antioxidant action is the most important. It enables them to prevent the onset of several degenerative diseases and disorders by scavenging free radicals, absorbing potentially hazardous light, and inhibiting the oxidation of lipids, proteins, and DNA (Bernstein et al., 2016). Table 5 provides a summary of the primary carotenoids' most significant characteristics.

**TABLE 5:** The most important properties of the main carotenoids.

Components	Main source	Benefits	Limitations	References
β‐carotene	Carrots, squash, tomatoes, sweet potatoes, apricots, yams, spinach, mangoes, green plants, green peppers, and other fruits and vegetables	Having the highest pro‐vitamin A activity among carotenoids, this potent antioxidant scavenges free radicals and lowers cancer, cataracts, infections, heart disease, and many other chronic diseases It also maintains skin health, improves vision, fortifies immune systems, and has immune‐modulating properties	It breaks down easily in the absence of oxygen, heat, and light. It is medically exceedingly unstable due to the large number of bound double bonds that form after conjugation. Additionally, it is hydrophobic in fluid, has a very high boiling point, and crystallizes at room temperature, but has a low (and variable) bioavailability through oral ingestion (less than 10% of food consumed orally)	Ahmad, Riaz, Shahzaib Nadeem, et al. (2022), Rehman et al. (2020), Chen et al. (2022)
Lycopene	Found in high concentrations in pink grapefruit, tomatoes, papaya, pink guava, and watermelon, also in apricots, plums, barriers, carrots, red cabbage, green peppers, passion fruit, and a large number of red‐colored fruits, vegetables, and microorganisms	The most powerful antioxidant across carotenoids is lycopene, having a structure with more conjugated double bonds than other carotenoids (lycopene from watermelon exhibited much better antimicrobial activity than from tomato) It boosts immunity, has anti‐inflammatory and immunomodulatory qualities, and prevents the growth of hepatic fibrogenesis, chronic heart disease, and certain types of cancer	Owing to the quantity of links in its molecular structure, it is extremely sensitive to oxygen, light, heat, and moisture in a biological sense. Additionally, it is unstable at basic pH, readily oxidized and isomerized, and destroyed at acidic pH. Its limited bioavailability due to its insoluble in water also prevents it from being incorporated into a wide range of foods and drinks	Sampaio et al. (2019), Lin et al. (2022), Shu et al. (2023)
Lutein	Widely distributed in plants, fungi, algae, and bacteria, especially in dark green leafy vegetables (spinach, broccoli, kale), orange‐yellow fruits and vegetables (petals of the marigold flower, carrots, honeydew melon, yellow corn, mango, and potatoes) and egg yolks	One of the largest concentrations of lutein (about 500 times greater than other tissues) is found in the retina. Additionally, it contains anti‐inflammatory, antioxidant, anti‐cancer, anti‐diabetic, anti‐obesity, anti‐aging, and anti‐fatigue qualities. Moreover, it prevents atherosclerosis and lowers the risk of prostate cancer	Relevance in the food and drug sectors is restricted by the fact that it is chemically volatile (degrades as a result of interactions with other substances in the food matrix; susceptible to light, heat, pH, and oxidative stress), solid at ambient temperature, very poor water solubility, and has limited bioavailability (some nutrients are very hydrophobic, and enzymatic processes and a low GIT pH additionally hinder absorption)	Xu et al. (2021)
Zeaxanthin	Egg, oranges, corn, honeydew melon, dark green leafy vegetables, bacteria	The macula contains lutein, which guards against damage to cells and genetic code, aging, burning, plaque in the arteries, better breathing, and general health loss from macular degeneration	Chemically unstable	Saini et al. (2022)

They constitute a sizable amount of our diet and can lessen the threat of evolving several chronic illnesses, including diabetes, cataracts, inflammatory processes, neural tube defects, coronary heart disease, and deformities of the muscles and brain. They can also reduce the risk of developing skin issues, Crohn's disease, celiac disease, and gastrointestinal disorders (Bernstein et al., 2016). Carotenoids are also known to participate in a variety of cell‐nuclear connections as well as DNA damage repair and anti‐damage mechanisms. Carotenoids are now widely used in the culinary, medical, cosmetic, and pharmaceutical sectors because of their well‐established health advantages and potent antioxidant properties (Ahmad, Riaz, Shahzaib Nadeem, et al., 2022; Kolašinac et al., 2021). This drug has a low bioavailability despite its numerous medical advantages. This is due to several factors. Food has to be consumed because people are not able to produce it. Second, because they are hydrophobic—that is, highly insoluble in water—the range of foods and drinks that can contain them is limited. Furthermore, fresh fruits and vegetables—which are thought to be the main food sources of carotenoids—have a limited bioavailability. According to Liu, Wang, et al. (2018), it varies from 10% to 20% for carotenes and up to 40% for xanthophylls.

Industries are shifting away from synthetic compounds due to the market's growing desire for natural colorants in the past few years. Carotenoids, along with betalain, chlorophyll, and anthocyanin, are among the most visually pleasing natural colorants. The main problem with carotenoids found in nature is that they are unstable and will oxidize when an oxidizing agent is present. Additionally, changes in pH, heating, and light exposure can modify the natural structure of carotenoids and the epoxidan extension of the polyene chain, all of which can impact bacterial activity (Janiszewska‐Turak, 2017).

The idea of solubility consists of two linked steps: Priority one is biological activity, followed by early bioaccessibility. Bioaccessibility is the amount of active molecules that are released from the food matrix, whereas biological activity is the movement of active substances toward the target locations. Carotenoids' bioavailability is frequently lowered due to their poor solubility, partial release from the food matrix, and potential for degradation during digestion (Soukoulis & Bohn, 2018). Systems for the effective and safe administration of carotenoids must be devised to address all of these concerns. Yet, for the project to be successful, the appropriate encapsulating materials and processes must be used. Therefore, encasing carotenoids in alginate matrices can boost their bioavailability and protect them from the gastrointestinal system's digestive enzymes (Liao et al., 2021). Thus, during use and storage, alginate‐based matrices ought to maintain their mechanical and chemical stability. Thus, alginate‐based matrices should retain their chemical and mechanical stability during usage and storage.

### 5.3 Encapsulation

The development of efficient carriers for providing bioactive substances to humans in the right amounts and the reduction of their beneficial properties during processing owing to oxidation, degradation, evaporation, or thermal denaturation are two of the main challenges. Among the most effective approaches to encapsulate unstable macromolecules is to wrap their active sections into thin films or trap them throughout the matrix using an array of edible coating/entrapping chemicals (Eun et al., 2020). An ideal encapsulation technique should lessen the sensitivity of environment‐sensitive active chemicals (such as carotenoids) and help carry them to the right location in the body for release. Moreover, hydrophilic and dissolving carotenoid‐loaded carriers are needed to maintain and improve the efficacy over time of carotenoids (Huang et al., 2021). The three main methods used to encapsulate carotenoids to improve their water dissolution, mechanical and chemically stable storage, bioavailability, and bioactivity, or to achieve their regulated and long‐term absorption, are lipid packaging, polymeric carrier packaging, and combinations of these. Table 6 summarizes the effects of several alginate‐based encapsulation systems on key carotenoids that are currently being studied.

**TABLE 6:** The impact of alginate‐based encapsulating systems on the properties of carotenoids.

Constituents	Encapsulation system	Benefits	Limitations	References
	Dried alginate beads	Increased stability, especially for the lowest alginate level, for storage under low‐oxygen and dark conditions		
β‐carotene	Alginate beads	Greater chemical resistance in the GIT, especially when there is more alginate present The beads have less alginate. Enhanced stability during storage (avoided degradation) more efficiently than if the particles contained more alginate	Free lipid droplets had higher bioaccessibility than hydrogel beads	
	Whey protein isolate (WPI)‐alginate‐chitosan capsule		WPI‐alginate chitosan capsule coating inhibits bioaccessibility	
	Alginate o/w emulsions	Enhanced chemical stability	Decrease bioaccessibility	
	Whey proteins and HPMC‐reinforced alginate hydrogels template by emulsions	Compared to employing only plain alginate particles, encapsulation efficiency is improved when whey proteins and HPMC are added to alginate gel		
	Oil droplets in caseinate/alginate microparticles	More chemical stability and bioaccessibility		
	Emulsion gels based on egg yolk granules and sodium alginate	Improved storage stability	Decreased bioaccessibility	Hasnain et al. (2020)
	Alginate‐pectin‐whey protein isolate stabilized emulsion	High encapsulation efficiency Stabilized Improved thermal, physical, and chemical stability were demonstrated by the emulsion. Additionally, it demonstrated significant release in the intestines and low production in the gut after digestion		Ye et al. (1991)
Lycopene	Alginate‐based emulsion gels containing protein‐coated droplets	Higher storage stability and bioaccessibility, faster release during in vitro digestion		Lin et al. (2022)
	Alginate beads with added sugars and galactomannans	Lycopene release strongly influenced by beads composition		Dos Santos et al. (2018)
	Alginate beads with modified rice starch	Increased stability compared to emulsion and solution		Sun et al. (2022)

Hydrogels such as starch and alginate, polymer complexes, liposomes as micelles, Pickering emulsions, and oil‐in‐water emulsions (multilayer, multiple, and oil‐in‐water emulsions) are commonly used to disperse carotenoids (Liu, Wang, et al., 2018). Carotenoids were best encapsulated by polymer compounds and micellar emulsions (Boonlao et al., 2022; Maghsoudi et al., 2022; Roll Zimmer et al., 2022). One especially useful kind of lipid‐based encapsulated technique for encasing and protecting carotenoids is fluids. Jin et al. (2023) claim that emulsion systems, yet, don't seem to have much control over how the parts they have coated are released and kept. Alginate‐based hydrogels are used as carrier matrices to encapsulate hydrophobic and hydrophilic active compounds in a variety of industries, such as biotechnology, food service, and biomedical engineering (Pajic‐Lijakovic et al., 2019). Because of its biological compatibility, affordability, mechanical strength under a variety of experimental conditions, along with suitable rheological behavior and ease of manipulation and processing into a variety of shapes and dimensions, alginate is becoming recognized as a good option for several applications (Milivojevic et al., 2015). However, a few practical restrictions apply to Ca‐alginate gels: big pore size, limited mechanical strength, low cryo‐ and dehydro‐capacities, and active component leakage. Usually, they are dealt with by mixing them with additional biopolymers. Ca‐alginate gels also have excellent carotenoid expression rates and a mild, aesthetically pleasing aqueous‐based gel formation, which are important for sensitive chemicals (Aguirre Calvo & Santagapita, 2019).

In alginate carriers, carotenoids are consistently more stable, but their bioaccessibility is reduced. Reduced bioaccessibility as a consequence of blocked active chemical flow via the gel matrix is the main reason for prolonged release. The impact is either nonexistent or, in some cases, even the opposite effect—an increase in bioaccessibility—is demonstrated in the context of micro and nanoparticles. Gel‐emulsified methods could also provide similar results. Encapsulation has two effects as a result.

## 6 CONCLUSION

The biological activity of polysaccharides generated from brown algae is influenced by various factors such as their size, conformation, configuration, location, molecular weight, monosaccharide composition, and amount of functional groups. Thus, categorizing polysaccharide structures and studying their biology and structure may disclose the molecular underpinnings of action and give the theoretical foundation for screening polysaccharides with intestine‐protective properties. Since disruptions are often associated with the formation of intestinal illnesses, intestinal protection requires maintaining the integrity of the intestinal barrier. The intestinal biological barrier is the most important of these for maintaining gut health. Intestinal microbiota and its metabolites may impact the structural integrity of the chemical barrier, epithelial barrier, and immune response. Much of the current research on intestinal microbiota has been on the amount of both beneficial and detrimental intestinal bacteria, the effects of medications and disease on microbiota, and the repercussions of focusing on microbiota on disease. Because of its various benefits, alginate and alginate hydrogels have been widely employed to encapsulate a variety of bioactive chemicals, including carotenoids. It is challenging to use them because of their limited absorption in the human body, water solubility, susceptibility to deterioration, and other characteristics. Encasing carotenoids in suitable delivery systems, such as alginate‐based carriers, can make them stable, soluble, and releasable under controlled circumstances. This keeps their beneficial properties and increases their bioavailability. This review can help scientists in a range of fields who work with carotenoids encapsulated within alginate‐based carriers to improve the packed method for producing carotenoids and recommend the best solutions for these systems.

## AUTHOR CONTRIBUTIONS

**Arslan Ahmad:** Conceptualization (equal); investigation (equal); resources (equal); supervision (equal). **Sakhawat Riaz:** Investigation (equal); writing – review and editing (equal). **Derese Tamiru Desta:** Conceptualization (equal); investigation (equal); methodology (equal); resources (equal); supervision (equal); validation (equal); writing – review and editing (equal).

## FUNDING INFORMATION

The authors declare that no funds, grants, or other support were received during the preparation of this manuscript.

## CONFLICT OF INTEREST STATEMENT

The authors declare that they have no known competing financial interests or personal relationships that could have appeared to influence the work reported in this paper.

## Data Availability

All authors state that further data will be provided upon request, even though sufficient data have been provided in the form of tables and figures.
